# FHOD1 and FMNL1 formin proteins in intestinal gastric cancer: correlation with tumor-infiltrating T lymphocytes and molecular subtypes

**DOI:** 10.1007/s10120-021-01203-7

**Published:** 2021-06-11

**Authors:** Naziha Mansuri, Vanina D. Heuser, Eva-Maria Birkman, Minnamaija Lintunen, Annika Ålgars, Jari Sundström, Raija Ristamäki, Olli Carpén, Laura Lehtinen

**Affiliations:** 1 Institute of Biomedicine and FICAN West Cancer Centre, University of Turku and Turku University Hospital, Kiinamyllynkatu 10, 20520 Turku, Finland; 2 Department of Pathology, Turku University Hospital and University of Turku, Kiinamyllynkatu 10, 20520 Turku, Finland; 3 Medicum, Research Program in Systems Oncology and HUSLAB, University of Helsinki and Helsinki University Hospital, Haartmaninkatu 3, 00014 Helsinki, Finland; 4 Department of Oncology, University of Turku and Turku University Hospital, Kiinamyllynkatu 4-8, 20521 Turku, Finland

**Keywords:** Gastric cancer, Formins, FHOD1, FMNL1, T lymphocytes

## Abstract

**Background:**

Gastric cancer (GC) is the third most common cause of cancer death. Intestinal type GC is a molecularly diverse disease. Formins control cytoskeletal processes and have been implicated in the progression of many cancers. Their clinical significance in GC remains unclear. Here, we characterize the expression of formin proteins FHOD1 and FMNL1 in intestinal GC tissue samples and investigate their association with clinical parameters, GC molecular subtypes and intratumoral T lymphocytes.

**Methods:**

The prognostic significance of FHOD1 and FMNL1 mRNA expression was studied with Kaplan–Meier analyses in an online database. The expression of FHOD1 and FMNL1 proteins was characterized in GC cells, and in non-neoplastic and malignant tissues utilizing tumor microarrays of intestinal GC representing different molecular subtypes. FHOD1 and FMNL1 expression was correlated with clinical parameters, molecular features and T lymphocyte infiltration. Immunohistochemical expression of neither formin correlated with survival.

**Results:**

Kaplan–Meier analysis associated high FHOD1 and FMNL1 mRNA expression with reduced overall survival (OS). Characterization of FHOD1 and FMNL1 in GC cells showed cytoplasmic expression along the actin filaments. Similar pattern was recapitulated in GC tissue samples. Elevated FMNL1 was associated with larger tumor size and higher disease stage. Downregulation of FHOD1 associated with TP53-mutated GC tumors. Tumor cell FHOD1 expression strongly correlated with high numbers of tumor-infiltrating CD8 + lymphocytes.

**Conclusions:**

FHOD1 and FMNL1 proteins are expressed in the tumor cells of intestinal GC and significantly associate with clinical parameters without direct prognostic significance. FHOD1 correlates with high intratumoral CD8 + T lymphocyte infiltration in this cohort.

**Supplementary Information:**

The online version contains supplementary material available at 10.1007/s10120-021-01203-7.

## Introduction

Gastric cancer (GC) is the fifth most common cancer type globally and the third most common cause of cancer death. Despite a few notable advances in the systemic management of GC, the overall prognosis of patients with metastatic disease remains poor [1]. Intestinal type GC is a heterogeneous and complex disease; it is the GC type with the most molecular diversity [2]. The new molecular classification of gastric cancer provides a vital insight knowledge on the molecular and biological behavior of gastric cancer [3, 4]. GC has been recognized as a collection of various molecularly driven particular entities rather than a single disease. Among these molecular GC subtypes, the most immunogenic tumors are the Microsatellite instable (MSI) and Epstein-Barr Virus positive (EBV +) GC subtypes. The MSI and EBV + GC has been studied extensively for the possibility of the utilization of targeted immunotherapy [5].

Formins are molecular scaffolds that nucleate actin by a pathway distinct from Arp2/3 complex, linking signal transduction to actin reorganization and gene transcription. Despite the significant discoveries on formins’ role in cell biology and embryogenesis, few studies have directly implicated their role in disease pathogenesis and tumor progression in general, and in gastric cancer in particular. Formin homology two domain-containing protein one or FHOD1, is a crucial regulator of cellular actin dynamics [6]. Previous studies by us and others implicate FHOD1 as an essential participant in cancer cell migration, invasion, and stress fiber formation [7–10]. FHOD1 has been implicated as one of the crucial genes linked with advanced disease and metastasis in GC patients [11]. In addition, FHOD1 is frequently overexpressed in triple-negative breast cancers [7]. The mechanisms that regulate formin expression are poorly known but may involve signaling pathways derailed in cancers. Human leukocyte formin or FMNL1 is mainly expressed in lymphoid tissues, such as the spleen and thymus, and the haematopoietic tissues; in addition, it is overexpressed in human hematological malignancies, more specifically in non-Hodgkin lymphoma and in leukemic cell lines [12–14]. FMNL1 is crucial for cellular processes in T lymphocytes and macrophages [15, 16]. The possible relationship between FMNL1 and FHOD1 with the tumor immune infiltrating lymphocytes in GC is still largely unstudied.

Here, we analyzed the expression of FHOD1 and FMNL1 in intestinal-type tumor samples of the stomach, gastrointestinal junction and distal esophagus and investigated their association with clinical data, including GC molecular subtypes. In addition, we characterized FHOD1 and FMNL1 expression in cultured gastric cancer cells. Our study is the first one to characterize these formins in intestinal gastric cancer tissue samples and investigate the correlation between cancer cell-specific FHOD1 and FMNL1 protein expression and intratumoral T lymphocyte infiltration.

## Materials and methods

### Analysis of mRNA data from public databases

The prognostic significance of FHOD1 and FMNL1 mRNA expression in gastric tumors was assessed by utilizing publicly available online database km-plotter (www.kmplot.com) [17]. Kaplan–Meier survival analyses were done for intestinal gastric cancer samples (*n* = 320), cut-off value was 291 (range 82–1706) for FHOD1 (probe: 218530_at) and 108 (range 2–1050) for FMNL1 (probe: 204789_at).

### Gastric cancer cell lines

Gastric cancer cell lines AGS, MKN28, and MKN45 were cultured in DMEM (Lonza, Basel, Switzerland) containing 10% fetal bovine serum (Biowest, Nuaillé, France), supplemented with 5 mM ultraglutamine and 100 U/ml penicillin–streptomycin (Gibco, CA, USA). The AGS cell line was derived from the primary gastric cancer of a 54-year-old female which exhibited characteristics of both Lauren subtypes [18]. The MKN28 cell line was derived from a lymph node metastasis of a 70-year-old female with a well-differentiated primary gastric cancer of intestinal histology [19]. The MKN45 cell line was derived from the liver metastasis of a 62-year-old patient with a poorly differentiated primary gastric cancer of diffuse histology [19].

### Western blotting

Western blot samples from gastric cancer cell lines were collected and processed as described elsewhere [18]. The rabbit anti-human FHOD1 or FMNL1 (1:1000, Sigma-Aldrich, St. Louis, MO) antibody was incubated overnight at 4 °C. Rabbit polyclonal to GAPDH—HRP conjugated (Abcam, Cambridge, UK) was used 1:5000 as a control for protein loading. The secondary antibodies were HRP-conjugated swine anti-rabbit and HRP-conjugated rabbit anti-mouse immunoglobulins (1:3000, Dako, Glostrup, Denmark). Membranes were washed three times with TBST between the different steps.

### Cell immunofluorescence staining and microscopy

Cells were plated on gelatin (Sigma-Aldrich) precoated coverslips (13 mm) and grown in complete medium for 24 h. The cells were fixed and stained as described in Peippo et al*.* [20]. Primary rabbit anti-human FHOD1 or FMNL1 antibodies (1:200, Sigma-Aldrich) were incubated for 1 h at RT. Secondary antibodies were Alexa Fluor 568 goat anti-rabbit IgG (1:500, Invitrogen, Carlsbad, CA). The filamentous actin was visualized with Alexa Fluor 488-conjugated phalloidin (1:500, Invitrogen). The mounting media contained DAPI for staining the nuclei (ProLong^®^ Gold Antifade Mountant with DAPI, Life Technologies). For negative controls, the cells were stained using secondary and phalloidin antibodies only. Images were taken with a Nikon Elipse Ni fluorescence microscope (Nikon Instruments).

### Patients and tumor specimens

The collection and characteristics of the study cohort have been previously reported [21]. In brief, a total number of 190 patients with intestinal-type gastric adenocarcinomas were selected out of a consecutive series of 244 patients diagnosed with adenocarcinoma of the stomach, gastro-esophageal junction (GOJ) or distal esophagus at the Turku University Hospital between years 1993 and 2012. For confirmation of diagnosis and adequacy of material, all corresponding haematoxylin–eosin (H&E) stained slides were reviewed. Tumor stage was assessed according to the current TNM classification manual [22]. The relevant clinical information was collected from the medical records. The median follow-up time was 125 months. Among these patients, 6.8% (13/190) received preoperative chemotherapy. Helicobacteria pylori status was available for 78/190 patients of which 20/78 were positive for *H. pylori*.

The intestinal-type cancers were classified based on the following criteria: EBER in situ hybridization positive tumors were classified as EBV-positive, tumors showing a complete loss of nuclear reactivity of at least one of the mismatch repair protein (MMR) markers (MLH1, MSH2, MSH6, PMS2) were classified as mismatch repair-deficient (MMR-D) and tumors with complete loss of or strong diffuse TP53 nuclear immunoreactivity were classified as TP53 aberrant. Tumors showing none of these alterations were classified as “other” [21]. 186 tumors were eligible for molecular classification. The reporting of the study has been performed following the current recommendations [23]. The study cohort characteristics are summarized in Table 1.

**Table 1:** Patient characteristics of the intestinal-type esophagogastric adenocarcinomas

Number of patients	*n* (%)
All	190
Median age at diagnosis (range)	74.4 (32.9–90.9)
Patient sex	
Female	68 (35.8)
Male	122 (64.2)
Site of primary tumor	
Distal oesophagus	19 (10.0)
GOJ/cardia	60 (31.6)
Corpus	52 (27.4)
Antrum/pylorus	59 (31.1)
Tumor differentiation grade	
Grade 1	17 (8.9)
Grade 2	93 (48.9)
Grade 3	80 (42.1)
Stage	
I	40 (21.1)
II	79 (41.6)
III	61 (32.1)
IV	10 (5.3)
Follow-up status	
Alive and free of disease	34 (17.9)
Alive with disease	1 (0.5)
Deceased	155 (81.6)
Molecular subtypes^a^	
EBV +	17 (9.1)^b^
MMR-D	19 (10.2)
TP53 aberrant	103 (55.4)
Others	52 (28.0)

^*a*^*MMR*-*D* mismatch repair deficient, *MMR*-*P* mismatch repair proficient, *EBV* Epstein–Barr virus

^b^The groups were not mutually exclusive. The percentages are calculated as a proportion of the 186/190 tumors eligible for molecular characterization

### Immunohistochemistry (IHC) of FHOD1 and FMNL1 formin expression

The expression of formins FHOD1 and FMNL1 in clinical gastric cancer samples was studied utilizing a ngTMA (next-generation tissue microarray). TMA construction has been described previously [21]. Briefly, whole slide images of representative tumors were sectioned at 4 µm, H&E stained, scanned and uploaded into a web portal (casecenter.utu.fi) for annotation. Four individual cores (1.0 mm in diameter) were collected from each tumor, two from the central area and two from the invasive front. In addition, normal gastric mucosa was included. FHOD1 and FMNL1 staining was performed according to the streptavidin-peroxidase method using a Labvision staining device (Thermo Fisher Scientific, Fremont, CA). Rabbit anti-human polyclonal monospecific antibodies were used (FHOD1 (HPA024468), dilution 1:150, Sigma-Aldrich, St Louis, MA and FMNL1 (20466-1-AP), dilution 1:500), Proteintech, Chicago, IL. Sample cores with less than 25% of tumor tissue were excluded from scoring. FMNL1 and FHOD1 stainings were scored as 0 (negative), 1 (weak), 2 (intermediate), or 3 (strong). Examples of the stainings are presented in Fig. 3b.

### Statistical analyses

The intensity of FHOD1 and FMNL1 expression was analyzed for association with clinical variables and differences among the molecular subtypes using *χ*2 test or Fisher’s exact test. Kaplan–Meier log-rank test was used for univariate survival analyses. Only recurrences ≥ 6 months after the time of diagnosis were considered relevant for the recurrence-free survival (RFS) which was calculated from the time of diagnosis to the time of first recurrence, death of any cause, or to the last follow-up date. Overall survival (OS) was calculated from the time of diagnosis to the time of death of any cause or the last follow-up date. Correlation of FHOD1 and FMNL1 expression to the number of lymphocytes in the same tissue samples were analyzed with one-way ANOVA or independent samples *T* test (with Levene’s test). Statistical analyses were performed with IBM SPSS Statistics for Windows, version 27.0 (IBM Corporation, Armonk, NY). *P* values < 0.05 were considered statistically significant.

## Results

### High FHOD1 and FMNL1 mRNA expression associate with poor overall survival in intestinal gastric cancer

**Fig. 1:**
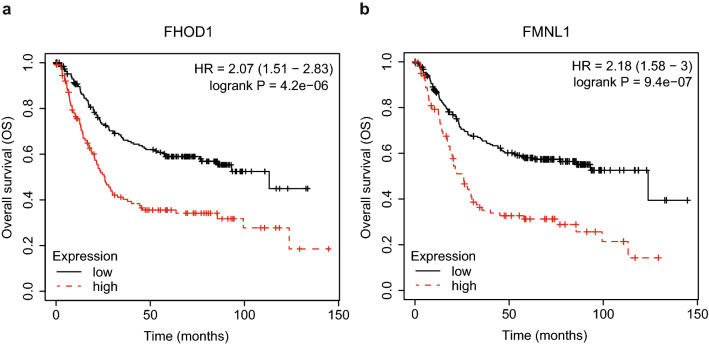
Prognostic significance of FHOD1 and FMNL1 mRNA expression in intestinal gastric cancer samples. **a** Kaplan–Meier plot (OS) of FHOD1 mRNA expression. **b** Kaplan–Meier plot (OS) of FMNL1 mRNA expression

To assess the prognostic significance of FHOD1 and FMNL1 expression in intestinal gastric cancer, we used the KM-plotter database for Kaplan–Meier survival analyses [17]. The overall survival (OS) of patients was significantly reduced with both high FHOD1 (HR 2.07, 95% CI 1.51–2.83, *p* = 4.2e–06) and high FMNL1 (HR 2.18, 95% CI 1.58–3, *p* = 9.4e–07) mRNA expression. The plots are presented in Fig. 1a, b, respectively. We also tested, whether correlation between FHOD1 and FMNL1 expression and survival was dependent on other relevant clinical parameters, but found that the variables did not affect the correlation significantly.

### Characterization of FHOD1 and FMNL1 expression in cultured gastric cancer cells

**Fig. 2:**
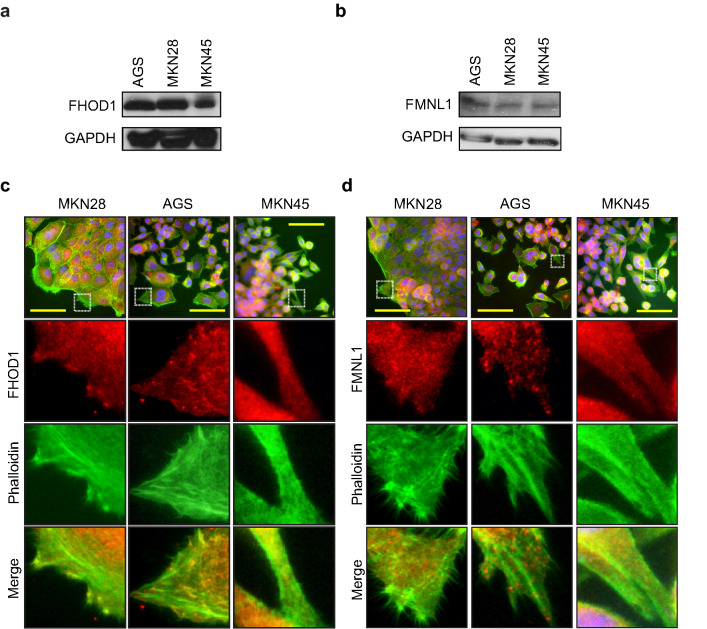
FHOD1 and FMNL1 expression and cellular localization in gastric cancer cell lines. **a**, **b** FHOD1 and FMNL1 expression levels in AGS, MKN28, and MKN45. GAPDH was used as a control for protein loading. **c**, **d** FHOD1 and FMNL1 cellular localization and expression pattern in GC cells. Actin filaments are stained with Alexa Fluor 488-conjugated phalloidin. Scale bars: 20 µm

To characterize FHOD1 and FMNL1 expression in gastric cancer cell lines and validate antibodies for tissue stainings, we studied FHOD1 and FMNL1 protein expression in three publicly available cell lines; MKN28, AGS and MKN45. All studied cell lines displayed intermediate to high expression of FHOD1, and low expression of FMNL1 (Fig. 2a, b, respectively). We did not detect any distinct differences in the expression levels between the different cell lines. To study the localization and expression pattern of these formins in gastric cancer cells, we performed double immunofluorescence staining of F-actin and FHOD1 or FMNL1 in MKN28, AGS and MKN45 cells (Fig. 2c, d, respectively). FHOD1 was expressed mostly in a dot-like pattern in the cytoplasm and along the actin filaments of all cell lines. FMNL1 distribution was cytoplasmic for all studied cell lines. Negative staining controls showed no unspecific staining (Supplementary Fig. S1).

### Expression of FHOD1 and FMNL1 in the non-neoplastic gastric mucosal lining and clinical samples of intestinal gastric cancer

**Fig. 3:**
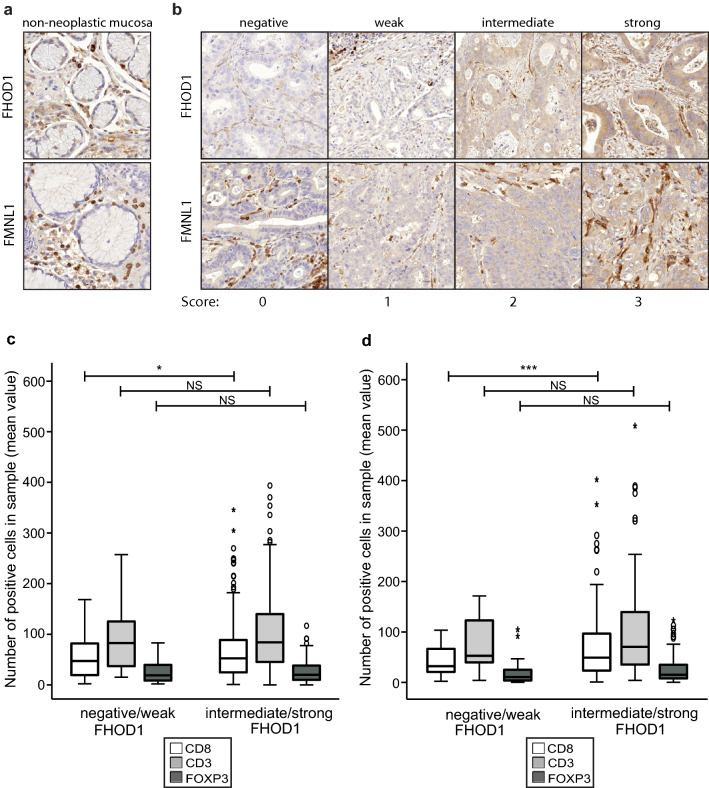
Immunohistochemical staining of FHOD1 and FMNL1 and association of high FHOD1 expression and intratumoral lymphocyte infiltration in intestinal gastric cancer. **a** Examples of FMNL1 and FHOD1 stainings in non-neoplastic gastric mucosa. **b**. Examples of FHOD1 and FMNL1 staining intensities and scoring. **c**, **d**: Analysis of correlation between FHOD1 expression and number of infiltrated lymphocytes (mean value) in central (**c**) and peripheral (**d**) tumor parts. The boxes refer to quartile distribution (25–75%) range, with the median value shown as a vertical line. Statistical significance is indicated with stars: *p < 0.05; **p < 0.01; ***p < 0.005

To determine the baseline staining intensities of FHOD1 and FMNL1, we studied non-neoplastic controls of gastric mucosa included in the TMAs. FHOD1 and FMNL1 staining intensity were low or negative in non-neoplastic gastric epithelium (Fig. 3a). The endothelium and plasma cells displayed positive staining for FHOD1, whereas lymphocytes, stromal cells and muscle cells stained positive for FMNL1. Based on these observations, we subsequently regarded the endothelial staining and plasma cells as internal positive controls for FHOD1 stainings in the tumor samples, and lymphocytes and muscle cells were, respectively, regarded as internal positive controls for FMNL1. In cancer cells, FHOD1 and FMNL1 showed different intensities of cytoplasmic expression. The stainings were scored as 0 (negative), 1 (weak), 2 (intermediate) or 3 (strong) (Fig. 3b). The expression of FHOD1 could be assessed in 177 (93%) cases, while FMNL1 could be assessed in 184 (96%) cases in the same TMAs.

### Correlation of FHOD1 and FMNL1 expression with clinical variables among intestinal gastric cancer subtypes

To assess the clinical significance of FHOD1 and FMNL1 expression in intestinal-type gastric cancer, we studied the correlation between clinical variables such as tumor size, stage and tumor location as well as molecular tumor subtypes (TP53, MMR and EBV status) and FHOD1 and FMNL1 expression. The FHOD1 stainings were categorized in two groups: (1) negative/weak and (2) intermediate/strong, while FMNL1 stainings were categorized in three groups: (1) negative/weak, (2) intermediate and (3) strong. The results are presented in Table 2, significant *p* values are indicated in bold. Intermediate FMNL1 expression in the peripheral tumor part associated significantly with higher tumor size (T3: 43% and T4: 36.7%, *p* = 0.023) and samples with high FMNL1 expression were exclusively either T3 or T4 tumors (50%/50%). There was also a similar trend with FHOD1 expression in the central tumor part, but this association was not statistically significant (T3: 39.3% and T4: 35%, *p* = 0.069). In addition, elevated FMNL1 expression correlated significantly with tumor stage (*p* = 0.004) regardless of the sample location (central or peripheral). Tumors with intermediate FMNL1 expression were primarily either stage II or stage III (43.6% and 35.6%, respectively) and tumors with high FMNL1 expression were mostly stage II or stage IV tumors (54.5% and 27.3%, respectively). Tumors with negative FHOD1 expression in their central part were significantly associated with mutated TP53 (77.1%, *p* = 0.007), while FMNL1 expression was not significantly associated to any of the molecular subtypes. We also studied the prognostic significance of FHOD1 and FMNL1, but neither of these formins showed significant association with disease outcome (OS or RFS) in this patient cohort.

**Table 2:** Correlation of the expression of FHOD1 and FMNL1 with clinical variables

	FMNL1 central				FMNL1 peripheral				FMNL1 strongest^a^				FHOD1 central			FHOD1 peripheral			FHOD1 strongest^a^		
	Neg/weak	Interm	Strong	*p* value	Neg/weak	Interm	Strong	*p* value	Neg/weak	INTERM	Strong	*p* value	Neg/weak	Interm./strong	*p* value	Neg/weak	Interm./strong	*p* value	Neg/weak	Interm./strong	*p* value
T																					
T1	11 (13.4)	5 (7.0)	0 (0.0)	0.700	11 (21.6)	3 (3.8)	0 (0.0)	**0.023**	10 (18.9)	6 (5.9)	0 (0.0)	0.128	2 (5.7)	14 (12.0)	0.069	1 (6.3)	12 (10.1)	0.919	1 (6.3)	15 (10.2)	0.789
T2	15 (18.3)	10 (14.1)	1 (14.3)		10 (19.6)	13 (16.5)	0 (0.0)		11 (20.8)	16 (15.8)	1 (9.1)		9 (25.7)	16 (13.7)		2 (12.5)	22 (18.5)		2 (12.5)	25 (17.0)	
T3	33 (40.2)	31 (43.7)	4 (57.1)		19 (37.3)	34 (43.0)	3 (50.0)		20 (37.7)	44 (43.6)	5 (45.5)		18 (51.4)	46 (39.3)		8 (50.0)	47 (39.5)		9 (56.3)	59 (40.1)	
T4	23 (28.0)	25 (35.2)	2 (28.6)		11 (21.6)	29 (36.7)	3 (50.0)		12 (22.6)	35 (34.7)	5 (45.5)		6 (17.1)	41 (35.0)		5 (31.3)	38 (31.9)		4 (25.0)	48 (32.7)	
Stage																					
I	22 (26.8)	12 (16.9)	0 (0.0)	**0.032**	19 (37.3)	12 (15.2)	0 (0.0)	**0.033**	19 (35.8)	17 (16.8)	0 (0.0)	**0.004**	8 (22.9)	26 (22.2)	0.750	2 (12.5)	30 (25.2)	0.636	2 (12.5)	34 (23.1)	0.660
II	30 (36.6)	30 (42.3)	5 (71.4)		18 (35.3)	33 (41.8)	3 (50.0)		16 (30.2)	44 (43.6)	6 (54.5)		16 (45.7)	44 (37.6)		8 (50.0)	43 (36.1)		6 (37.5)	58 (39.5)	
III	27 (32.9)	26 (36.6)	0 (0.0)		11(21.6)	30 (38.0)	2 (33.3)		16 (30.2)	36 (35.6)	2 (18.2)		10 (28.6)	40 (34.2)		5 (31.3)	38 (31.9)		7 (43.8)	47 (32.0)	
IV	3 (3.7)	3 (4.2)	2 (28.6)		3 (5.9)	4 (5.1)	1 (16.7)		2 (3.8)	4 (4.0)	3 (27.3)		1 (2.9)	7 (6.0)		1 (6.3)	8 (6.7)		1 (6.3)	8 (5.4)	
Location																					
Distal oesophagus	9 (11.0)	8 (11.3)	1 (14.3)	0.817	7 (13.7)	10 (12.7)	0 (0.0)	0.500	6 (11.3)	11 (10.9)	1 (9.1)	0.713	5 (14.3)	13 (11.1)	0.378	3 (18.8)	13 (10.9)	0.151	1 (6.3)	17 (11.6)	0.509
GOJ/cardia	28 (34.1)	25 (35.2)	2 (28.6)		13 (25.5)	32 (21.5)	2 (33.3)		15 (28.3)	39 (38.6)	3 (27.3)		13 (37.1)	36 (30.8)		6 (37.5)	42 (35.3)		5 (31.3)	50 (34.0)	
Corpus	19 (23.2)	21 (29.6)	3 (42.9)		15 (29.4)	17 (21.5)	3 (50.0)		15 (28.3)	24 (23.8)	5 (45.5)		11 (31.4)	30 (25.6)		6 (37.5)	29 (24.4)		7 (43.8)	37 (25.2)	
Antrum/pylorus	26 (31.7)	17 (23.9)	1 (14.3)		16 (31.4)	20 (25.3)	1 (16.7)		17 (32.1)	27 (26.7)	2 (18.2)		6 (17.1)	38 (32.5)		1 (6.3)	35 (29.4)		3 (18.8)	43 (29.3)	
P53																					
Mutant–type	51 (62.2)	37 (52.1)	4 (57.5)	0.465	32 (62.7)	46 (59.0)	4 (66.7)	0.909	35 (66.0)	54 (54.0)	6 (54.5)	0.347	27 (77.1)	60 (51.3)	**0.007**	11 (68.8)	67 (56.8)	0.362	13 (81.3)	80 (54.8)	**0.042**
Wild-type	31 (37.8)	34 (47.9)	3 (42.9)		19 (37.3)	32 (41.0)	2 (33.3)		18 (34.0)	46 (46.0)	5 (45.5)		8 (22.9)	57 (48.7)		5 (31.3)	51 (43.2)		3 (18.8)	66 (45.2)	
MMR																					
MMR-D	10 (12.2)	4 (5.6)	0 (0.0)	0.252	5 (9.8)	5 (6.4)	0 (0.0)	0.697	6 (11.3)	8 (8.0)	0 (0.0)	0.591	1 (2.9)	13 (11.1)	0.191	0 (0.0)	10 (8.5)	0.608	0 (0.0)	14 (9.6)	0.364
MMR-P	72 (87.8)	67 (94.4)	7 (100)		46 (90.2)	73 (93.6)	6 (100)		47 (88.7)	92 (92.0)	11(100)		34 (97.1)	104 (88.9)		16 (100)	108 (91.5)		16 (100)	132 (90.4)	
EBV																					
Positive	5 (6.1)	6 (8.5)	2 (28.6)	0.112	4 (7.8)	4 (5.1)	1 (16.7)	0.361	3 (5.7)	8 (8.0)	3 (27.3)	0.090	2 (5.7)	10 (8.5)	0.734	0 (0.0)	12 (10.2)	0.359	1 (6.3)	13 (8.9)	< 0.999
Negative	77 (93.9)	65 (91.5)	5 (71.4)		47 (92.2)	74 (94.9)	5 (83.3)		50 (94.3)	92 (92.0)	8 (72.7)		33 (94.3)	107(91.5)		16 (100)	106 (89.8)		15 (93.8)		

Statistically significant *p* values are indicated in bold

*MMR*-*D* mismatch repair deficient, *MMR*-*P* mismatch repair proficient, *EBV* Epstein–Barr virus

^a^Sample with the strongest intensity, central or peripheral

### Association of FHOD1 and FMNL1 expression with intratumoral lymphocyte infiltration

In our previous study, we stained immune cell markers CD3, CD8 and FOXP3 from the same ngTMA utilized in this study, and saw that high intratumoral infiltration with CD3- and CD8-positive lymphocytes was significantly associated with better outcome [24]. To find out whether there was any association of FHOD1 or FMNL1 expression with intratumoral lymphocyte infiltration in these TMA samples, we tested the correlation between FHOD1 and FMNL1 expression and the number of CD3-, CD8- and FOXP3-positive lymphocytes in individual samples. The analyses showed that tumors with intermediate or high tumor cell FHOD1 staining intensity harbored significantly higher numbers of CD8-positive cells (*p* = 0.039 in central and *p* = 0.003 in peripheral part) than tumors with negative or weak FHOD1 expression (Fig. 3c, d, respectively, and Table 3). A similar trend was seen with CD3 + T lymphocytes in central tumor samples, but this observation was not statistically significant (*p* = 0.090). Samples with very high average CD8 + and CD3 + numbers (outliers) were exclusively among tumors with intermediate or high FHOD1 expression. Tumor cell FMNL1 expression did not correlate with lymphocyte infiltration.

**Table 3:** Association of FHOD1 tumor cell expression with tumor lymphocyte infiltration

	Central tumor sample					Peripheral tumor sample				
	*N*	Mean	Min	Max	*p value*	*N*	Mean	Min	Max	*p value*
CD8 + average										
FHOD1 neg/weak	40	53.5	2.7	168.5	**0.039**	19	42.5	2.1	103.5	**0.003**
FHOD1 interm/high	143	71.7	0.8	345.6		132	71.7	0.9	402.0	
CD3 + average										
FHOD1 neg/weak	40	85.9	15.2	257.3	0.090	19	70.8	4.0	171.4	0.216
FHOD1 interm/high	143	104.9	2.5	429.9		132	96.4	4.0	508.0	
FOXP3 average										
FHOD1 neg/weak	40	25.1	2.0	83.0	0.614	19	22.5	0.2	104.8	0.708
FHOD1 interm/high	143	27.2	0.3	116.7		133	24.9	0.3	123.0	

Statistically significant *p* values are indicated in bold

## Discussion

The crucial contribution of formins to cell migration and invasion is well established in many cancers, but their expression and function in gastric cancer (GC) remains mostly uncovered. Here, we characterized the expression patterns of FHOD1 and FMNL1 formins in clinical samples of non-neoplastic gastric tissue and intestinal GC and in GC cell lines, and investigated their potential clinical significance. We focused here on intestinal-type tumors only, as their biologic origin is distinct from the diffuse type gastric cancer. Our study cohort includes also tumors from the gastro-esophageal junction and distal esophagus, which share the molecular and morphological characteristics of intestinal-type gastric cancer, especially the TP53 mutant (CIN) and the EBV-/TP53WT/MMR-proficient (other) subtypes [4, 20].The expression was correlated with important clinical variables and different molecular subtypes of intestinal GC. In addition, we analyzed the correlation of FHOD1 and FMNL1 expression with intratumoral T lymphocyte infiltration in the same samples. To our knowledge, this study is the first to utilize clinical tissue samples in examining the role of FMNL1 and FHOD1 in intestinal-type GC.

Recently, elevated expression of FMNL1 and FMNL3 mRNAs were associated with poor prognosis and immune infiltration in GC in a study by Nie et al*.* [25]. The analyses were conducted using several publicly available online databases containing mRNA expression data. We found similar association of high FHOD1 and FMNL1 mRNA expression with poor outcome using an online database km-plotter [17]. However, when assessing the cancer cell-specific FHOD1 and FMNL1 protein expression in intestinal GC tissue samples, we did not find direct prognostic significance (OS or PFS) for high FHOD1 or FMNL1 expression. As FHOD1 and FMNL1 are prominently expressed in lymphocytes and macrophages in certain tissues [16, 26], we hypothesize that this difference may arise from technical issues. RNA analyses are typically performed from tissue bulk containing not only cancer cells but also mesenchymal cells, including FHOD1 and FMNL1 expressing immune cells. This can lead to an overestimation of the cancer cell-specific mRNA expression, while in our approach, we only analyzed cancer cell-specific FHOD1 and FMNL1 staining and disregarded the strongly stained immune cells from our scoring results. Nie et al*.* also state in their report that high formin mRNA expression negatively correlated with tumor purity, indicating that in tumors with high FMNL1 expression, less cancer cells were present [25].

We further characterized FHOD1 and FMNL1 protein expression levels and patterns in gastric cancer cells. We chose cell lines representing well differentiated (MKN28) and poorly differentiated (AGS, MKN45) GC. The cell stainings showed typical dot-like expression patterns and cytoplasmic location along the actin filaments, which have been previously reported in other cancer cell types [7, 8, 20]. This cytoplasmic pattern was recapitulated in the gastric cancer tissue samples, further confirming the integrity of the immunohistochemical stainings. Non-neoplastic tissue samples displayed negative or weak expression of both FHOD1 and FMNL1. FHOD1 was strongly expressed in endothelium and plasma cells, whereas strong FMNL1 was seen in lymphocytes, stromal spindle cells and muscle cells. Using these as internal positive controls for the tumor sample stainings, we were able to enhance the quality of our analysis and confirm the tumor cell-specific scoring.

Importantly, even though no direct prognostic significance was seen, both FHOD1 and FMNL1 expression associated with other clinical parameters. Elevated expression of FMNL1 was associated with larger tumor size and with more advanced disease stage. Also, FHOD1 expression was elevated in larger tumors, but statistical significance was not achieved. These results indicate that FMNL1 may have a role in GC tumor progression. A similar association has been seen in other cancers [8, 27].

We have previously described an easily adaptable method for identifying distinct molecular subtypes among intestinal gastric cancer, based on immunohistochemical and in situ hybridization markers [21]. In this study we analyzed the association of FHOD1 and FMNL1 expression with the molecular subtypes and found that TP53-mutated subtype was associated with negative or weak FHOD1 staining. This indicates a possible down-regulation of FHOD1 in TP53-mutated tumors. One of the pathways altered in TP53-mutated GC is the phosphatidylinositol-3-kinase (PI3K–AKT) [4], which is essential in the control of FHOD1 expression [10]. While the mechanism governing the connection between TP53 and FHOD1 remains unclear, there are mutual links indicating the need for further investigation.

Our previous study with the same GC cohort showed that increased numbers of intratumoral CD3 + and CD8 + T lymphocytes were associated with a favorable outcome [24]. Here, we wanted to investigate whether intratumoral T lymphocyte infiltration and formin expression in the same tissue samples are interconnected. Indeed, we found a correlation between tumor cell FHOD1 expression and high numbers of CD8 + lymphocytes. TP53-mutated GC tumors generally display lower intratumoral lymphocyte infiltration and wild-type TP53 tumors, in turn, harbor high numbers of infiltrating lymphocytes [24]. We found down-regulation of FHOD1 in TP53 aberrant tumors and upregulation of FHOD1 in tumors with high lymphocyte infiltration. In contrast to a previous finding [25], such association was not seen with tumor cell FMNL1 expression and number of infiltrated lymphocytes.

Although prognostic significance for the studied formins could not be recapitulated in immunohistochemical analyses, the study describes novel data on formin expression, cellular localization and possible correlation with immune cell recruitment in intestinal GC. Furthermore, our results demonstrate a correlation between FMNL1 expression and tumor size as well as tumor stage, suggesting that FMNL1 may play a role in tumor progression in intestinal GC.

## Supplementary Information

Below is the link to the electronic supplementary material.

Supplementary Figure S1. Negative staining controls for FHOD1 and FMNL1 immunofluorescence stainings (DOCX 437 KB)
