# Supplementary figures and images for: FHOD1 and FMNL1 formin proteins in intestinal gastric cancer: correlation with tumor-infiltrating T lymphocytes and molecular subtypes
Source: Gastric Cancer. 2021 Jun 11;24(6):1254–63. doi: 10.1007/s10120-021-01203-7 (PMC8502136; doi:10.1007/s10120-021-01203-7)

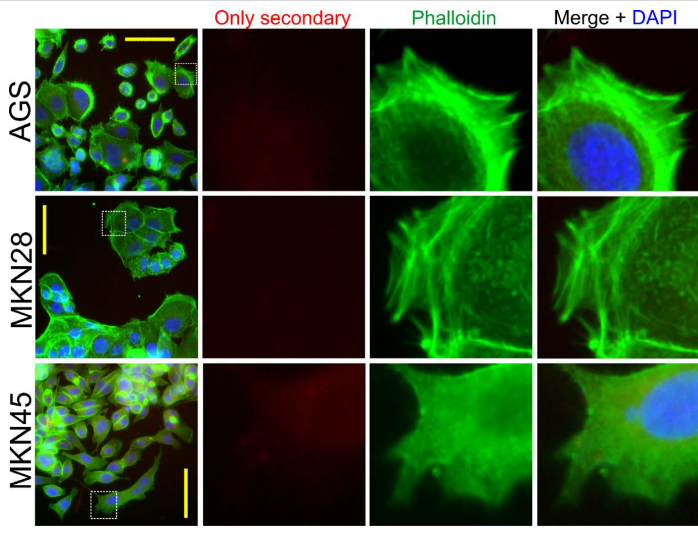

Supplement: Supplementary file 1 — Supplementary Figure S1. Negative staining controls for FHOD1 and FMNL1 immunofluorescence stainings (DOCX 437 KB) [file 10120_2021_1203_MOESM1_ESM.docx]
